# Adsorption Characteristics for Cu(II) and Phosphate in Chitosan Beads under Single and Mixed Conditions

**DOI:** 10.3390/polym15020421

**Published:** 2023-01-13

**Authors:** Taehoon Kim, Jeongwoo Shin, Byungryul An

**Affiliations:** Department of Civil Engineering, Sangmyung University, Cheonan 31066, Republic of Korea

**Keywords:** adsorption, chitosan, amino group, Cu(II), phosphate

## Abstract

Chitosan, a natural organic polymer, has shown bifunctional characteristics in the removal of cationic and anionic contaminants from water and wastewater treatment. In particular, cationic Cu(II) and anionic phosphate can simultaneously interact with chitosan owing to the presence of the amino group in the form of NH_2_ and NH_3_^+^ in chitosan. To gain greater insight into the bifunctional adsorption characteristics of chitosan, its adsorption capacity for Cu(II) and phosphate was tested under single and mixed (co-ion) conditions to investigate the interactions between four types of chitosan beads and NH_2_ and NH_3_^+^. In the single condition, Cu(II) uptake was reduced from 0.243 to 0.0197 mmol/g due to the crosslinking and drying processes, whereas no significant reduction in phosphate uptake was observed, indicating that the crosslinking agent only interacted with NH_2_ to decrease the number of available adsorption sites for Cu(II). Under the mixed condition, the simultaneous presence of the two ions clearly increased the uptake of each other, with the adsorption of phosphate being more influenced than that of Cu(II). The comparison of the rate constant, *k*_1_ or *k*_2_, using pseudo-first- and pseudo-second-order models confirmed that phosphate reached equilibrium faster than Cu(II), suggesting that electrostatic interaction was preferred over coordination. In addition, under mixed conditions, co-ion competition slowed down the adsorption kinetics for both Cu(II) and phosphate.

## 1. Introduction

Natural biopolymers such as chitosan, alginate, and glucose have been widely applied in the industrial field as an alternative to synthetic polymers due to their biocompatibility and nontoxicity to humans [1]. In particular, natural biopolymers are efficient adsorbents in separating cationic heavy metals such as Cu(II) [2], Cd(II) [3], and Cr(VI) [1] from water, showing high capacity when used in the form of beads.

Among them, chitosan is particularly attractive because it contains the amino functional group (NH_2_), which renders it suitable for water and wastewater treatment. Chitosan is obtained from chitin, which is extracted from the shells of crabs or shrimps. After deacetylation via base treatment (NaOH) [4], the acetyl group of chitin is converted to amino (NH_2_) groups to produce chitosan. The degree of deacetylation (DD) is considered a critical parameter to determine the number of amino groups in chitosan [5]; specifically, a higher DD affords more amino groups, thereby increasing the number of available adsorption sites [6].

Chitosan shows complexation ability with heavy metals, which decreases in the order Cu > Hg > Zn > Cd > Ni > Co [7]. This affinity order is similar to that of the commercial chelating resin Chelex 100, having iminodiacetic acid groups [8], a newly developed chelating resin with carboxylate acid groups [9], and XFS 4195 containing bis-picolylamine [10], indicating that the fixed chelating ligands selectively remove metal ions. Therefore, the NH_2_ species of chitosan acts as a fixed chelating ligand and interacts with heavy metals via coordination.

Furthermore, the anion removal efficiency can be enhanced by adding a polymeric ligand exchanger (PLE), which was first introduced by Helfferich (1962) [11]. The adsorbed metal ions are immobilized and serve as adsorption sites for anionic contaminants by participating in Lewis acid–base interactions.

Meanwhile, at pH values below the pK_a_ of chitosan, the NH_2_ group is protonated into NH_3_^+^, which can remove anions such as phosphate [12,13] and arsenic [14] by forming electrostatic interactions. Moreover, phosphate (pK_a1_ = 2.1, pK_a2_ =7.2, and pK_a3_ = 12.3) existed as four different species (H_3_PO_4_, H_2_PO_4_^−^, HPO_4_^2−^, and PO_4_^3−^) depending on the pH [15]. Therefore, the degree of protonation plays a critical role in determining the fraction of NH_2_ and NH_3_^+^ and, in turn, the adsorption mechanism and capacity for cations and anions.

Hydrogel beads were used to obtain high adsorption capacity when chitosan was introduced as an adsorbent in water treatment and wastewater treatment. However, its application has been limited due to its physical strength and chemical stability. Hydrogel beads are easily broken under acidic conditions and relatively long operation periods. As a result, crosslinking and drying processes have been added to increase the stability and strength, with the possibility of changing adsorption properties. Therefore, other types of CBs have been more frequently applied in this field [16,17].

In this study, aiming at gaining more insight into the bifunctional adsorption characteristics of the amino group of chitosan, the removal of Cu(II) as a cationic metal and phosphate as an anionic ligand was investigated using four types of chitosan beads (CB) prepared via crosslinking and drying. Specifically, the objectives of this work were (1) to calculate the capacity of the four types of CB for Cu(II) and phosphate under single and mixed (co-ion) conditions, (2) to evaluate the effect of the initial concentration of Cu(II) and phosphate on the adsorption capacity for both ions, and (3) to determine the adsorption kinetics of coordination and electrostatic interaction using two kinetic models and compare the value of the rate constants *k*_1_ and *k*_2_ in the equilibrium.

## 2. Material and Methods

### 2.1. Chemicals

Medium molecular weight chitosan powder (~250,000 g/mol) with a DD of 75–85% was obtained from Sigma-Aldrich (St. Louis, MO, USA). A glutaraldehyde (GA) solution (25%) was purchased from SHOW (Tokyo, Japan) and used as a crosslinking agent. HCl, NaOH, CuCl∙2H_2_O, and KH_2_PO_4_ were purchased from Sigma-Aldrich (St. Louis, MO, USA). All chemicals were used as received without further purification.

### 2.2. Preparation of Four Types of CB

First, hydrogel CB (HCB) was prepared by dissolving chitosan flakes in acid. Then, CB were obtained under basic conditions [18]. The preparation conditions were dependent upon the molecular weight and DD of chitosan. Briefly, following the report by Shin et al. (2021) [19], a 2.5% (*w*/*w*) chitosan solution was prepared by dissolving the chitosan flakes in 1% HCl solution. Subsequently, the chitosan solution was added dropwise into a 1 mol/L NaOH solution. The mixture was mildly stirred for at least 12 h to complete the formation of CB, which was then washed until the solution pH was 7.5–8.0. The as-obtained HCB were subjected to chemical treatment (crosslinking) using a 0.5 mol/L GA solution to produce HCB-G. Both HCB and HCB-G were dried under air, affording the dried samples DCB and DCB-G, respectively.

### 2.3. Batch Removal Test

The removal experiments were performed in batch mode. Typically, a 50 mL solution containing ~20 mg/L (0.315 mmol/L) of Cu(II) and 30 mg/L (0.315 mmol/L) of phosphate for the mixed condition experiments and 20 mg/L of Cu(II) or 30 mg/L of phosphate for the single condition experiments was placed in a 55 mL conical tube. Another batch test was conducted using HCB-G to examine the effect of the initial concentration. The concentrations of Cu(II) and phosphate were set to 20, 40, and 60 mg/L and 30, 60, and 90 mg/L, respectively, which are expressed as the molar ratio of 1, 2, and 3 in the results. A batch kinetic test was conducted using DCB under single and mixed conditions with 20 mg/L of Cu(II) and 30 mg/L phosphate in 1 L of solution. The initial pH was set to 5.25 ± 0.3. All experiments were repeated at least twice and the initial pH was adjusted using HCl or NaOH during the experiment.

### 2.4. Adsorption Isotherm Studies

Langmuir [20] and Freundlich [21] isotherm equations were used to calculate the adsorption amount according to Equations (1) and (2), respectively:

(1) $$q_{e}=\frac{QbC_{e}}{1+bC_{e}}$$

(2) $$q_{e}=k_{f}C_{e}^{1/n}$$

where *q_e_* and *Q* are the adsorbed amount of target contaminant and the maximum uptake of contaminant per unit mass of adsorbent (mg/g), respectively, *C_e_* is the equilibrium concentration (mg/L) in solution, *b* is the Langmuir constant, and *k_f_* and *n* are the Freundlich constants.

### 2.5. Kinetic Test

Since DCB clearly showed adsorption behavior under single and mixed conditions, it was selected to determine the distinct adsorption rate in the liquid and solid phases using pseudo-first-order (PFO) and pseudo-second-order (PSO) kinetic models according to Equation (3), with *n* = 1 [22] and 2 [23,24], respectively.

(3) $$\frac{dq_{t}}{dt}=k_{n}{\left(q_{e}-q_{t}\right)}^{n}$$

The coefficient of determination *R*^2^ and the difference between experimental and calculated uptake (*χ*^2^; Equation (4)) were used to identify the best-fitting model.

(4) $$\chi^{2}=\sum \frac{{\left(q_{e,exp}-q_{e,cal}\right)}^{2}}{q_{e,cal}}$$

### 2.6. Chemical Analyses

Inductively coupled plasma spectroscopy (Model: NexION 300D, Agilent Technologies, Santa Clara, CA, USA) was used to determine the concentration of Cu(II) and phosphorus (as phosphate) in the solution. A Laboratory VIS spectrophotometer (Model: DR3900, HACH, USA) for Cu(II) and a portable analyzer (Model: HS-1000plus, HUMAS, Daejeon, Korea) for phosphorus were used as auxiliary equipment. The solution pH was measured using an ORION Star A22 pH meter (Thermo Scientific, Waltham, MA, USA).

## 3. Results and Discussion

### 3.1. Batch Removal Experiments Using Four Types of CB

**Figure 1:**
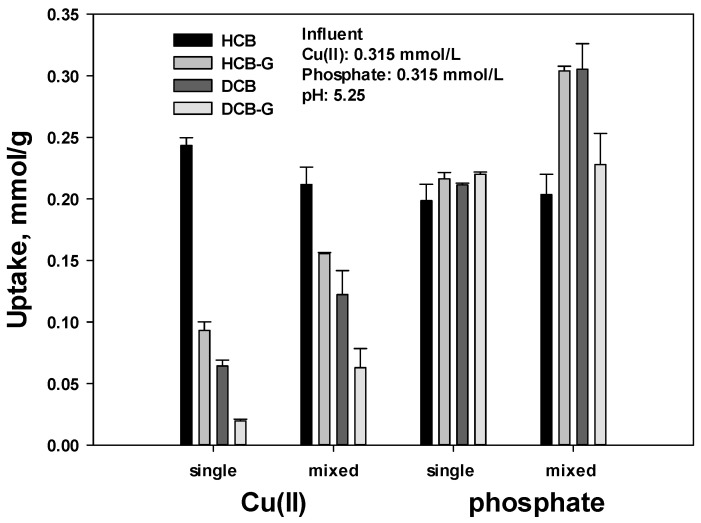
Cu(II) and phosphate uptake (mmol/g) using four types of chitosan beads (CB), namely hydrogel CB (HCB), glutaraldehyde-treated HCB (HCB-G), and the corresponding dried samples of DCB and DCB-G, under single and mixed conditions.

Figure 1 shows the uptake in mmol/g of Cu(II) and phosphate using four types of CB, i.e., HCB, HCB-G, DCB, and DCB-G, under single and mixed conditions. The adsorption uptake and behavior for Cu(II) and phosphate were obviously different depending on the type of CB. Under the single condition, the uptake of Cu(II) was 0.243, 0.0932, 0.0642, and 0.0197 mmol/g for HCB, HCB-G, DCB, and DCB-G, respectively, whereas the uptake of phosphate did not depend significantly on the type of CB and was in the range 0.199–0.220; thus, the phosphate uptake was 2.32, 3.29, and 11.4 times higher than that of Cu(II), except for HCB. This indicates that the interaction of the crosslinking agent GA, which was used to increase the chemical stability of the chitosan polymer and the drying process that strengthens the physical hardness, influences, to a larger extent, the uptake of Cu(II) rather than phosphate. The reaction between chitosan and GA was described in a previous study [23]. In solution, GA is composed of the following species: free aldehyde (I), monohydrate (II), dehydrate (III), cyclic cis (IV), and trans (V) isomers. These species interact with the deprotonated amino group (NH_2_) of the chitosan polymer and not with the protonated amino group (NH_3_^+^) [25,26]. The drying of hydrogel chitosan decreases the porous volume and pore size resulting in a reduction in adsorption capacity and adsorption kinetics for cationic heavy metals [17]. The uptake of phosphate as an anion was not significantly altered upon drying. As a result, the drying serves only to destroy the NH_2_ site. Therefore, the use of GA and drying reduce the number of available NH_2_ sites for Cu(II) adsorption.

The total uptake (Cu(II) and phosphate) of 0.442 (0.243 + 0.198) mmol/g for HCB under a single condition would correspond to the maximum amount of available amino groups involved in electrostatic and coordination interactions under the present experimental conditions. This means that only the number of amino groups (NH_2_ or NH_3_^+^) would be affected by the experimental conditions, resulting in a change in the uptake of Cu(II) and phosphate. The fact that no significant change in phosphate uptake occurred under a single condition indicates that NH_2_ was not converted to NH_3_^+^, confirming that NH_2_, and not NH_3_^+^, was involved in the crosslinking and drying processes.

The Cu/phosphate separation factor (SF, *α_Cu/P_*) was calculated using the following Equation (5) to provide the selectivity in a binary system:

(5) $$\alpha_{Cu/P}=\frac{q_{Cu}\cdot C_{P}}{C_{Cu}\cdot q_{P}}$$

where *q* and *C* represent the uptake and concentration in the solid phase (mmol/g) and the aqueous phase (mmol/L), respectively. When SF (*α_Cu/P_*) is greater than unity, the selectivity of the adsorbent (solid) for Cu(II) is higher than for phosphate. The SF value was 3.63, 0.443, 0.265, and 0.234 for HCB, HCB-G, DCB, and DCB-G, respectively. Thus, except for HCB, phosphate was preferred over Cu(II), confirming that the crosslinking and drying processes affected the adsorption.

Under the single condition, the maximum uptake by CB was 0.243 mmol/g for Cu(II) and 0.220 mmol/g for phosphate via coordination and electrostatic interactions, respectively. Meanwhile, the Cu(II) and phosphate uptakes were significantly increased under mixed conditions. This result suggests that an additional interaction occurred during the adsorption under mixed conditions. In general, the increased uptake of both Cu(II) and phosphate could be explained in terms of precipitation and coprecipitation, increased ionic strength, and additional interactions. To avoid the precipitation of metal hydroxide, the pH of the initial solution was adjusted, and a control sample (without adsorbent) subjected to the same experiments showed no notable difference in the results. With regard to the ionic strength, the value increases with the charge number of the ion regardless of whether it is positive or negative. The increased uptake under mixed conditions was influenced by the increased ionic strength [27]. Finally, an additional interaction could involve a metal–ligand interaction, as was first described by Helfferich [11]. In this case, under mixed conditions, the adsorbed Cu(II) ions firmly bound to N atoms would interact with phosphate via electrostatic interaction, resulting in metal–ligand complexation, which would contribute to the removal of the target phosphate ligand [28,29].

### 3.2. Effect of the Initial Concentration of Cu(II) and Phosphate

**Figure 2:**
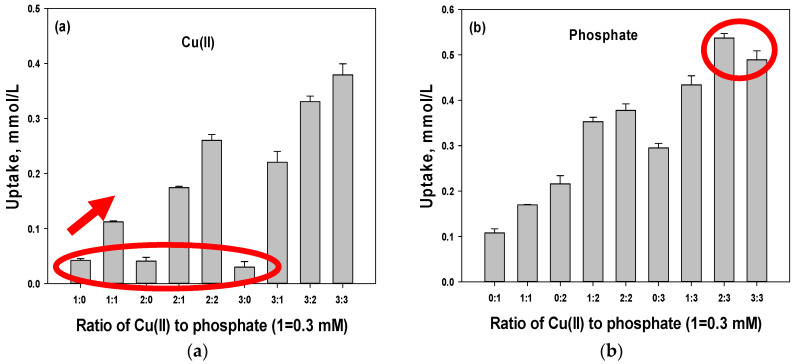
Uptake of Cu(II) (**a**) and phosphate (**b**) at different initial concentrations.

Since the uptake of Cu(II) and phosphate increased under mixed conditions, the effect of the initial concentration of Cu(II) and phosphate was investigated for DCB (Figure 2). The ratio of the X-axis represents the mole number (1 = ~0.315 mM). The following observations can be extracted from the results depicted in Figure 2: (1) under a single condition, as the initial concentration increased, the Cu(II) uptake did not increase (red circle in Figure 2a), but that of phosphate did (Figure 2b); (2) the uptake of Cu(II) and phosphate increased with increasing co-ion concentrations; (3) a decrease in phosphate uptake was observed at 3:3 (red circle in Figure 2b); and (4) the increase rate decreased with increasing co-ion concentrations.

To investigate whether these results were due to the effect of Cu(II) and phosphate species at increased initial concentrations, the species were calculated using Visual MINTEQ 3.1 at a fixed pH of 5.25 (Table 1). Since the dominant species of Cu(II) and phosphate were Cu^2+^ and H_2_PO_4_^−^ (94.3% and 93.7%, respectively), in all cases, the effect of the species could be ignored.

**Figure 3:**
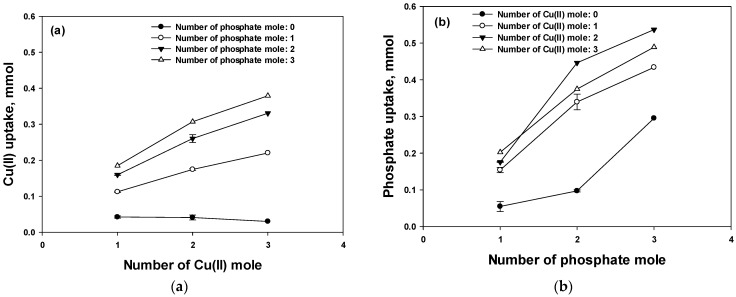
Uptake of Cu(II) (**a**) and phosphate (**b**) at different initial concentrations.

Since the Cu(II) uptake did not change in the absence of phosphate (Figure 3a, filled circles) and increased in the presence of 1, 2, and 3 mol of phosphate with a slope of 0.0540, 0.0855, and 0.0971 and an *R*^2^ of 0.978, 0.990, and 0.993, respectively, it can be concluded that NH_2_ in the polymer was already saturated with Cu(II) and the increased phosphate concentration led to a linear increase in the Cu(II) uptake, which could be explained by the formation of a Cu–phosphate complex with the addition of phosphate. In contrast, as shown in Figure 3b, the phosphate uptake increased in the absence of Cu(II) upon increasing the phosphate amount, which can be attributed to the strong driving force of the gradient overwhelming the mass transfer resistance. The phosphate uptake was increased in the presence of Cu(II) but was not significantly influenced by the increase in the Cu(II) concentration.

### 3.3. Adsorption Isotherm Studies

**Figure 4:**
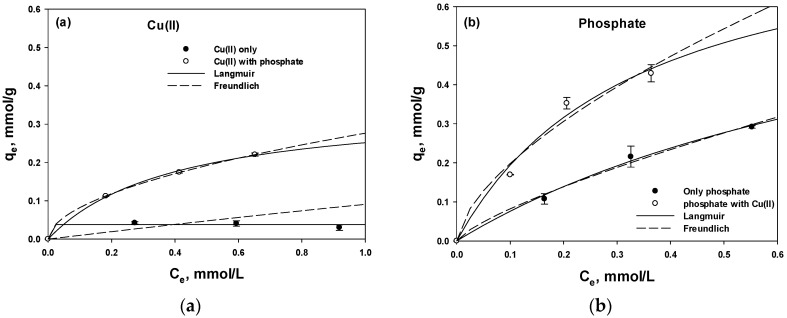
Langmuir and Freundlich isotherms for the adsorption of Cu(II) (**a**) and phosphate (**b**).

Next, to gain more insight into the adsorption mechanism, Langmuir and Freundlich’s isotherms were applied. The calculated parameters and fitting results are shown in Table 2 and Figure 4. In general, the adsorption mechanism is determined based on the best fit between the Langmuir and Freundlich models, which represent chemical and physical adsorption, respectively [30]. No significant difference was observed in the *R*^2^ values between Langmuir and Freundlich isotherms (Table 2), except for the Cu-single of Freundlich, suggesting that both electrostatic interaction and coordination occurred. A poor fitting of the Freundlich isotherm (*R*^2^ = 0.00431) to the Cu-single condition was observed. It is because the Freundlich isotherm model is better for unfavorable shapes when *n* is less than one (e.g., *n* < 1.00). Therefore, only four sample points did not satisfy the Freundlich observation.

Figure 4 clearly confirmed that the phosphate uptake was higher than the Cu(II) uptake. In the Langmuir and Freundlich isotherms, the *Q* and *k_f_* values of phosphate uptake increased from 0.820 to 0.850 (+3.66%) and from 0.466 to 0.840 (+80.2%), respectively, indicating that the presence of the co-ion in the system accelerated the adsorption capacity. The value of *b* in the Langmuir Equation (1) and *n* in the Freundlich Equation (2) represent the adsorption energy and the energetic heterogeneity of the adsorption sites, respectively, and are indicators of the adsorption affinity [31,32]. Thus, the increase in *b* from 1.02 to 2.97 and in *n* from 1.33 to 1.60 under mixed conditions confirmed that the adsorption was favored by the presence of the co-ion.

### 3.4. Adsorption Kinetics

A linear PFO kinetic model (Equation (6)) was obtained by integrating Equation (3) for *n* = 1 under the condition: *t* = 0, *q_t_* = 0, *t* = t, *q_e_* = *q_t_*. Then, a nonlinear PFO kinetic model (Equation (7)) can be obtained by rearranging Equation (6).

(6) $$ln\left(q_{e}-q_{t}\right)=lnq_{e}-k_{1}t$$

(7) $$q_{t}=q_{e}\left(1-e^{-k_{1}t}\right)$$

Similarly, linear and nonlinear PSO kinetic models were obtained from Equation (3) for *n* = 2 (Equations (8) and (9), respectively).

(8) $$\frac{t}{q_{t}}=\frac{1}{k_{2}q_{e}^{2}}+\frac{1}{q_{e}}t$$

(9) $$q_{t}=\frac{k_{2}q_{e}t}{1+k_{2}q_{e}^{2}t}$$

Generally, linear PFO and PSO kinetic models are first obtained and then the nonlinear models were rearranged to improve the correlation coefficient from linear models because the linear PFO model requires at least two segment lines separating two time conditions, i.e., the initial time and the rest time [33,34]. Therefore, the nonlinear PFO model is normally more appropriately used to explain the adsorption mechanism. Moreover, when the nonlinear kinetic model was converted to the linear kinetic model, model parameters of the nonlinear kinetic model would be distorted, and because the nonlinear kinetic equation does not need the value of *q_e,exp_* before fitting, it would be less affected by *q_e,exp_* [35,36].

**Figure 5:**
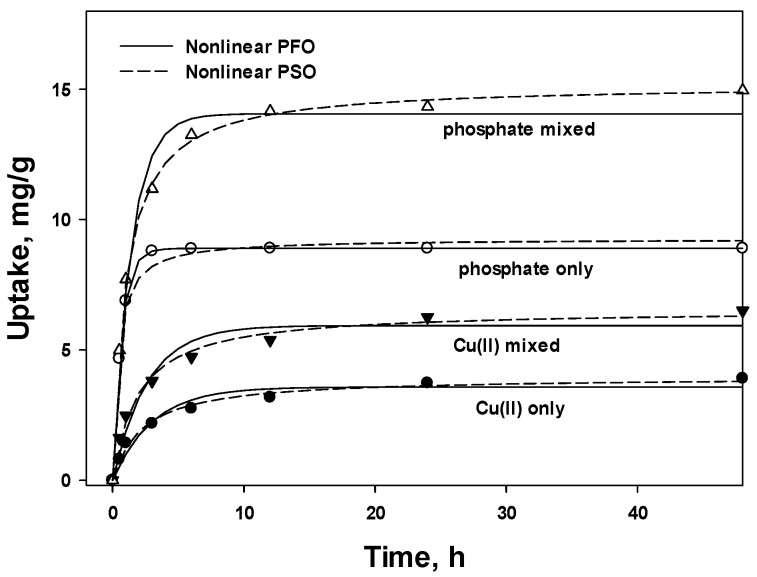
Nonlinear pseudo-first-order (PFO) and pseudo-second-order (PSO) kinetics of Cu(II) and phosphate under single and mixed conditions.

Nonlinear PFO and PSO kinetic models are plotted in Figure 5, and the calculated parameters *q_e_*, *k*, *χ*^2^, and *R*^2^ are listed in Table 3. As shown in Figure 5, the equilibrium was reached faster for the PFO kinetic model than for the PSO kinetic model. The profile of phosphate under single and mixed conditions was much sharper at the initial stages and reached equilibrium earlier than that of Cu(II) under both conditions, indicating that the adsorption of phosphate was faster than that of Cu(II), as can be extracted by comparing the *k*_1_ and *k*_2_ values. In general, the PSO model showed higher *R*^2^ and lower *χ*^2^ values than the PFO model, and no significant difference was observed between nonlinear and linear PSO models. As mentioned above, the lowest *R*^2^ is generally ascribed to the linear PFO model requiring two segments of time, and the higher fit of the PSO model suggests that chemical adsorption dominates over physical adsorption [37].

Under the single condition, *k*_1_ values of 0.328 and 1.49 were obtained for Cu(II) and phosphate, respectively, using the nonlinear PFO model and the corresponding *k*_2_ values of 0.112 and 0.277 for the PSO model. For both linear and nonlinear models, the adsorption rate for phosphate was almost four times or two times higher than that for Cu(II) using the PFO and PSO models, respectively, which is in accord with the sharper curve for phosphate observed in Figure 5. This indicates that the electrostatic interaction between phosphate and NH_3_^+^ was faster than coordination. Under the mixed condition, lower *k* values were obtained for both Cu(II) and phosphate compared with those under single conditions, except for Cu(II) in the PFO model, which can be disregarded because the PSO model afforded a better *R*^2^ than the PFO model. The lower *k* values can be attributed to the competition between co-ions regardless of their individual adsorption capacity [38,39]. Under the mixed condition, the adsorption rate for Cu(II) and phosphate were reduced to −28.9 and −76.2, and to −32.1 and −71.3 for nonlinear and linear PSO models, respectively. The reduction in the adsorption rate for phosphate was almost twice that of Cu(II) in the presence of the co-ion.

**Figure 6:**
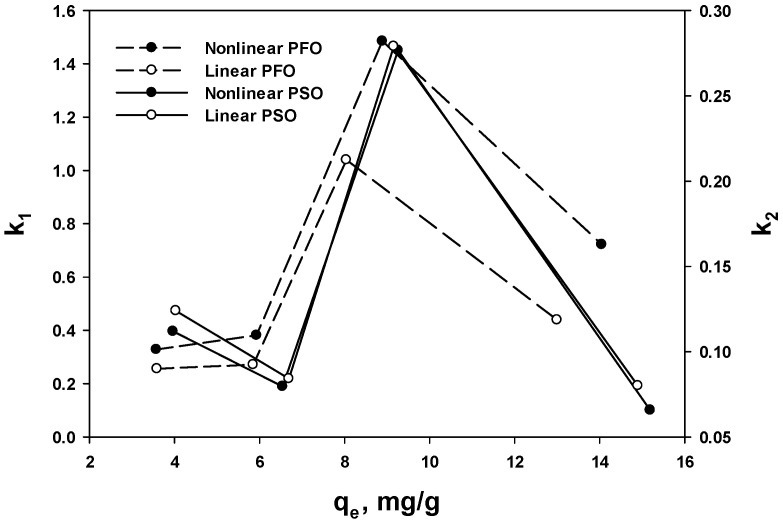
Plots of the adsorbed amount (*q_e_*) vs. rate constants *k*_1_ and *k*_2_.

In the PFO and PSO equations, *k* represents the rate constant, with higher *k* values indicating higher kinetics. To determine the effect of *k* on the equilibrium kinetics, the relationship between *k* and *q_e_* was examined. However, as shown in Figure 6, no relationship was found between *k*_1_ or *k*_2_ and *q_e_*. Similarly, previous studies using CB reported *q_e_* values of 118, 102, and 44.5 for *k* values of 0.382, 0.432, 0.384, respectively, and *q_e_* values of 7.97, 3.42, and 0.66 for *k* values of 0.17, 0.31, 0.23, respectively [40,41]. Therefore, as an alternative approach to evaluating the meaning of *k*, the adsorption kinetics were studied by plotting *q_t_*/*q_e_* vs. time, which would represent the time required to reach equilibrium regardless of the uptake.

**Figure 7:**
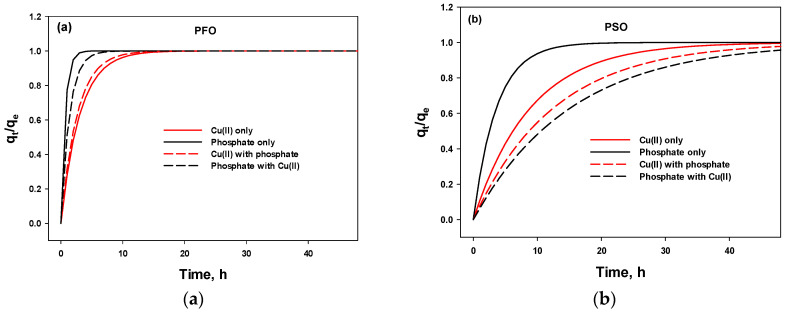
Plots of *q_t_*/*q_e_* versus time using pseudo-first-order (PFO) (**a**) and pseudo-second-order (PSO) kinetic models (**b**).

The values of *q_t_* and *q_e_* were obtained from the nonlinear PFO and PSO models. The results are shown in Figure 7 and Table 4. Table 4 summarizes the ratio of *q_t_* to *q_e_* (*q_t_*/*q_e_*), time, and the time ratio under single and mixed conditions. Regardless of the value of *q_t_*/*q_e_*, the time ratio was constant, which allowed for the direct comparison of the adsorption kinetics. In addition, the ratio of *k*_1_ or *k*_2_ was the same under single and mixed conditions. For example, 1.16 from 0.381 (*k*_1_, Cu(II)-single)/0.328 (*k*_1,_ Cu(II)-mixed), 0.485 from 0.722 (*k*_1_, phosphate-single)/1.49 (*k*_1_, phosphate-mixed), 0.711 from 0.0796 (*k*_2_, Cu(II)-single)/0.112 (*k*_2,_ Cu(II)-mixed), and 0.237 from 0.0657 (*k*_2_, phosphate-single)/0.277 (*k*_2_, phosphate-mixed), which are in accord with the results listed in Table 4. Therefore, *k*_1_ and *k*_2_ can be directly used to determine the adsorption kinetics and equilibrium time.

## 4. Conclusions

The mechanisms of the adsorption of Cu(II) and phosphate on CB were investigated in terms of the type of interaction, i.e., coordination and electrostatic interaction, between the adsorbate ion and the amino group in chitosan, which may exist as NH_2_ and NH_3_^+^ depending on the degree of protonation (α) and the solution pH. Under the single condition, the maximum uptake of Cu(II) and phosphate was 0.244 and 0.220 mmol/g, respectively. The NH_2_ and NH_3_^+^ species in CB were involved in the Cu(II) and phosphate adsorption, respectively.

The fact that a significant decrease in the Cu(II) uptake was observed depending on the type of CB, whereas no significant difference was detected in the phosphate uptake, indicated that the crosslinking agent introduced to increase the chemical stability interacted with NH_2_, thereby reducing the adsorptive capacity for Cu(II). The increased Cu(II) and phosphate uptake observed under mixed conditions using HCB-G and DCB was caused by an interaction between Cu(II) and phosphate. Upon increasing the initial concentration, the Cu(II) uptake increased proportionally with the phosphate concentration, whereas the increase in the phosphate uptake was less influenced by the Cu(II) concentration. The comparison of the rate constants *k*_1_ or *k*_2_ clearly showed that the electrostatic interaction was faster than coordination since the *k* value for phosphate adsorption was higher in both PFO and PSO models. In addition, the presence of the co-ion slowed down the adsorption kinetics. It was also shown that the *k*_1_ and *k*_2_ values can be directly used to determine the adsorption kinetics and equilibrium time by plotting *q_t_*/*q_e_* vs. time.

Based on the conclusions, further studies focusing on the pH are needed to quantitatively identify the adsorption characteristics and mechanisms of the amino group in chitosan. There were still some uncertainties regarding the removal amounts, such as mmol or mg quantities of cation and anion. For example, HCB did not show the effect of metal–ligand complexation. This is likely due to the change in the point of zero charge (pzc) of the chitosan bead or interface pH change, even though the solution pH was adjusted to 5.25 ± 0.3 at a desired time during the experiments. Various pH conditions determining the degree of protonation, the pzc, and the species of phosphate better clarify both electrostatic and coordination interactions. Additionally, the change in the pH of the solution would be investigated in metal complexation.

## Figures and Tables

**Table 1:** The portion of Cu(II) and H_2_PO_4_^−^ species at the different initial concentrations using Visual MINTEQ 3.1.

Species	Ratio of Cu(II) to Phosphate														
	1:0	0:1	1:1	2:0	0:2	2:1	1:2	2:2	3:0	0:3	3:1	1:3	3:2	2:3	3:3
Cu(II), %	99.5	-	97.6	99.5	-	97.8	95.6	95.9	99.4	-	97.6	94.1	95.9	94.3	94.6
H_2_PO_4_^−^, %	-	99.1	97.0	-	99.1	95.2	97.0	95.3	-	99.0	93.7	97.1	93.8	95.4	93.9

**Table 2:** The parameters were calculated from Langmuir and Freundlich isotherm models under single and mixed conditions.

Condition	Langmuir			Freundlich		
	*Q*	*b*	*R* ^2^	*k_f_*	*n*	*R* ^2^
Cu—single	0.0379	402,853	0.923	1.00	0.100	0.00431
Cu—mixed	0.353	2.48	0.999	0.277	1.90	1.00
Phosphate—single	0.820	1.02	0.995	0.466	1.33	0.992
Phosphate—mixed	0.850	2.97	0.985	0.839	1.60	0.975

**Table 3:** Parameters calculated from linear and nonlinear PFO and PSO models under single and mixed conditions.

Kinetic Model	Parameter	Cu(II) Single	Cu(II) Mixed	Phosphate Single	Phosphate Mixed	Average
Nonlinear PFO	*q_e_*, mg/g	3.57	5.93	8.89	14.1	
	*k*_1_	0.328	0.381	1.49	0.722	
	Δ*k*_1_, %		+16.2		−51.4	
	*R* ^2^	0.963	0.959	0.989	0.983	0.974
	*χ* ^2^	0.319	0.566	0.105	0.367	0.339
Linear PFO	*q_e_*, mg/g	3.59	5.85	8.04	13	
	*k*_1_	0.256	0.271	1.04	0.439	
	Δ*k*_1_, %		+5.86		−57.8	
	*R* ^2^	0.94	0.937	0.993	0.958	0.957
Nonlinear PSO	*q_e_*, mg/g	3.96	6.54	9.26	15.2	
	*k*_2_	0.112	0.0796	0.277	0.0657	
	Δ*k*_2_, %		−28.9		−76.2	
	*R* ^2^	0.992	0.991	0.98	0.999	0.991
	*χ* ^2^	0.0624	0.115	0.168	0.0174	0.0907
Linear PSO	*q_e_*, mg/g	4.03	6.69	9.16	14.9	
	*k*_2_	0.124	0.0842	0.279	0.0801	
	Δ*k*_2_, %		−32.1		−71.3	
	*R* ^2^	0.997	0.997	0.999	0.999	0.998

**Table 4:** Comparison of equilibrium time for different conditions and models.

PFO							PSO						
*q_t_*/*q_e_*, %	Cu(II)			Phosphate			*q_t_*/*q_e_*, %	Cu(II)			Phosphate		
	Single	Mixed	Ratio	Single	Mixed	Ratio		Single	Mixed	Ratio	Single	Mixed	Ratio
	Time, h	Time, h	Single/Mixed	Time, h	Time, h	Single/Mixed		Time, h	Time, h	Single/Mixed	Time, h	Time, h	Single/Mixed
30	1.09	0.935	1.17	0.240	0.494	0.486	30	3.19	4.48	0.712	1.29	5.43	0.238
50	2.12	1.82	1.16	0.467	0.959	0.4487	50	6.19	8.71	0.711	2.51	10.6	0.237
80	4.91	4.22	1.16	1.08	2.23	0.484	80	14.4	20.2	0.713	5.82	24.5	0.238
95	9.13	7.86	1.16	2.02	4.15	0.487	95	26.8	37.6	0.713	10.8	45.6	0.237
average			1.16			0.486				0.712			0.237

## Data Availability

The data used to support the findings of the study are available from the corresponding author upon request.

## References

[1] Sessarego S., Rodrigues S.C.G., Xiao Y., Lu Q., Hill J.M. (2019). Phosphonium-enhanced chitosan for Cr(VI) adsorption in wastewater treatment. Carbohydr. Polym..

[2] Patrulea V., Negrulescu A., Mincea M.M., Pitulice L.D., Spiridon O.B., Ostafe V. (2019). Optimization of the removal of copper(II) ions from aqueous solution on chitosan and cross-linked chitosan beads. Bioresources.

[3] Zhang F., Wang M., Zhou L., Ma X., Zhou Y. (2015). Removal of Cd(II) from aqueous solution using cross-linked chitosan–zeolite composite. Desalin. Water Treat..

[4] Anwar M., Anggraeni A.S., Al Amin M.H. (2017). Comparison of green method for chitin deacetylation. AIP Conf. Proc..

[5] Wang Q.Z., Chen X.G., Liu N., Wang S.X., Liu C.S., Meng X.H., Liu C.G. (2006). Protonation constants of chitosan with different molecular weight and degree of deacetylation. Carbohydr. Polym..

[6] Rhazi M., Desbrieres J., Tolaimate A., Rinaudo M., Vottero P., Alagui A. (2002). Contribution to the study of the complexation of copper by chitosan and oligomers. Polymer.

[7] Rinaudo M. (2006). Chitin and chitosan: Properties and applications. Prog. Polym. Sci..

[8] Hubicki Z., Kołodyńska D., Kilislioglu A. (2012). Selective removal of heavy metal ions from waters and waste waters using ion exchange methods. Ion Exchange Technologies.

[9] Jiang J., Ma X.-S., Xu L.-Y., Wang L.-H., Liu G.-Y., Xu Q.-F., Lu J.-M., Zhang Y. (2015). Applications of chelating resin for heavy metal removal from wastewater. e-Polymers.

[10] Grinstead R. (1984). Selective absorption of copper, nickel, cobalt and other transition metal ions from sulfuric acid solutions with the chelating ion exchange resin XFS 4195. Hydrometallurgy.

[11] Helfferich F. (1962). Ion Exchange.

[12] Jóźwiak T., Kowalkowska A., Filipkowska U., Struk-Sokołowska J., Bolozan L., Gache L., Ilie M. (2021). Recovery of phosphorus as soluble phosphates from aqueous solutions using chitosan hydrogel sorbents. Sci. Rep..

[13] Szymczyk P., Filipkowska U., Kuczajowska-Zadrożna M. (2016). Phosphate removal from aqueous solutions by chitin and chitosan in flakes. Prog. Chem. Appl. Chitin Deriv..

[14] Gérente C., Andrès Y., McKay G., Le Cloirec P. (2010). Removal of arsenic(V) onto chitosan: From sorption mechanism explanation to dynamic water treatment process. Chem. Eng. J..

[15] Snoeyink V.L., Jenkins D. (1980). Water Chemistry.

[16] Pakdel P.M., Peighambardoust S.J. (2018). Review on recent progress in chitosan-based hydrogels for wastewater treatment application. Carbohydr. Polym..

[17] Ruiz M., Sastre A., Guibal E. (2002). Pd and Pt recovery using chitosan gel beads. I. Influence of the drying process on diffusion properties. Sep. Sci. Technol..

[18] Guibal E., Milot C., Tobin J.M. (1998). Metal-anion sorption by chitosan beads:  equilibrium and kinetic studies. Ind. Eng. Chem. Res..

[19] Shin J., Kim T., Lee Y., An B. (2021). The effect of crosslinking and dry for the adsorption rate on the chitosan bead. J. Korean Soc. Water Wastewater.

[20] Langmuir I. (1916). The constitution and fundamental properties of solids and liquids. J. Am. Chem. Soc..

[21] Freundlich H.M.F. (1906). Over the adsorption in solution. J. Phys. Chem..

[22] Lagergren S. (1898). About the theory of so-called adsorption of soluble substances. Sven Vetensk. Handingarl..

[23] Blanchard G., Maunaye M., Martin G. (1984). Removal of heavy metals from waters by means of natural zeolites. Water Res..

[24] Ho Y.S. (1995). Adsorption of Heavy Metals from Waste Streams by Peat. Ph.D. Dissertation.

[25] Kildeeva N.R., Perminov P.A., Vladimirov L.V., Novikov V.V., Mikhailov S.N. (2009). About mechanism of chitosan cross-linking with glutaraldehyde. Russ. J. Bioorg. Chem..

[26] Monteiro C., Airoldi O.A.C. (1999). Some studies of crosslinking chitosan–glutaraldehyde interaction in a homogenous system. Int. J. Biol. Macromol..

[27] Cai W., Navarro D.A., Du J., Ying G., Yang B., McLaughlin M.J., Kookana R.S. (2022). Increasing ionic strength and valency of cations enhance sorption through hydrophobic interactions of PFAS with soil surfaces. Sci. Tot. Environ..

[28] Zhao D., Sen Gupta A.K. (1998). Ultimate removal and recovery of phosphate from wastewater using a new class of polymeric exchangers. Water Res..

[29] An B., Jung K.Y., Lee S.H., Lee S., Choi J.W. (2014). Effective phosphate removal from synthesized wastewater using copper-chitosan bead: Batch and fixed-bed column studies. Water Air Soil Pollut..

[30] Ho Y.S., Porter J.F., McKay G. (2002). Equilibrium isotherm studies for the sorption of divalent metal ions onto peat: Copper, nickel and lead single component systems. Water Air Soil Pollut..

[31] Na C.-K., Han M.-Y., Park H.-J. (2011). Applicability of theoretical adsorption models for studies on adsorption properties of adsorbents. J. Korean Soc. Environ. Eng..

[32] Treybal R.E. (1981). Mass-Transfer Operations.

[33] Hamdaoui O. (2006). Batch study of liquid-phase adsorption of methylene blue using cedar sawdust and crushed brick. J. Hazard. Mater..

[34] Moussout H., Ahlafi H., Aazza M., Maghat H. (2018). Critical of linear and nonlinear equations of pseudo-first order and pseudo-second order kinetic models. Karbala Int. J. Mod. Sci..

[35] Lin J., Wang L. (2009). Comparison between linear and non-linear forms of pseudo first-order and pseudo-second-order adsorption kinetic models for the removal of methylene blue by activated carbon. Front. Environ. Sci. Engin. China.

[36] Tran H.N., You S.-J., Hosseini-Bandegharaei A., Chao H.-P. (2017). Mistake and inconsistencies regarding adsorption of contaminants from aqueous solutions: A critical Review. Water Res..

[37] Magdy Y.H., Altaher H. (2018). Kinetic analysis of the adsorption of dyes from high strength wastewater on cement kiln dust. J. Environ. Chem. Eng..

[38] Lee C.H., Park J.M., Lee M.G. (2015). Competitive adsorption in binary solution with different mole ratio of Sr and Cs by aeolite A: Adsorption isotherm and kinetics. J. Environ. Sci. Int..

[39] Sun D., Zhang X., Wu Y., Liu T. (2013). Kinetic mechanism of competitive adsorption of disperse dye and anionic dye on fly ash. Int. J. Environ. Sci. Technol..

[40] Kurczewska J. (2022). Chitosan-montmorillonite hydrogel beads for effective dye adsorption. J. Water Process. Eng..

[41] Li H., Ji H., Cui X., Che X., Zhang Q., Zhong J., Jin R., Wang L., Luo Y. (2021). Kinetics, thermodynamics, and equilibrium of As(III), Cd(II), Cu(II) and Pb(II) adsorption using porous chitosan bead-supported MnFe_2_O_4_ nanoparticles. Int. Min. Sci. Technol..

